# Visual vertical neglect in acquired brain injury: a systematic review

**DOI:** 10.3389/fpsyg.2024.1360057

**Published:** 2024-03-11

**Authors:** Pasquale Moretta, Nicola Davide Cavallo, Eleonora Fonzo, Antonio Maiorino, Cesario Ferrante, Pasquale Ambrosino, Cinzia Femiano, Gabriella Santangelo, Laura Marcuccio

**Affiliations:** ^1^Istituti Clinici Scientifici Maugeri IRCCS, Neuromotor Rehabilitation Unit of Telese Terme Institute, Benevento, Italy; ^2^Department of Psychology, Università della Campania ‘Luigi Vanvitelli’, Caserta, Italy; ^3^Istituti Clinici Scientifici Maugeri IRCCS, Scientific Directorate of Telese Terme Institute, Benevento, Italy

**Keywords:** visual vertical neglect, visuospatial disorders, acquired brain injury, rehabilitation, clinical neuropsychology

## Abstract

Vertical neglect represents a visuospatial deficit occurring as a possible consequence of acquired brain injury (ABI). Differently from unilateral spatial neglect on horizontal space, vertical neglect is poorly studied in the literature and rarely assessed in clinical practice. In the available studies, the terms “radial,” “vertical,” and “altitudinal” neglect are often used interchangeably, although they do not describe the same spatial dimension. “Altitudinal” and “vertical” refer to the sagittal plane, whereas “radial” refers to the transverse plane. The term “vertical” is sometimes used interchangeably with respect to both axes. The aim of this systematic review was to identify the main characteristics of vertical neglect after ABI, the diagnostic tools used, and the treatment options. We also proposed a clarification of the manifestations and characteristics of vertical and radial neglect. The 23 articles reviewed, showed that the vertical neglect occurred more frequently on the lower space than on the upper space, that its presence was associated with horizontal neglect, and that it could also occur with compromise of the radial space, with the near radial being more common. The most frequent etiology associated with vertical neglect is vascular, particularly ischaemic. The lesions side are very heterogeneous and include both cortical and subcortical areas and all lobes, although the temporal lobe is most affected. With regard to the assessment tools, paper and pencil tasks are the most commonly used diagnostic tools to identify vertical neglect, although in recent years the use of computer-based tasks increased. Taken together, our results suggest that vertical neglect may be underestimated in patients with right hemisphere lesions and should always be assessed, especially in cases where the patient shows signs of horizontal neglect. The clinical assessment of vertical neglect is very important since it can lead to important functional limitations in everyday life, such as poor wheelchair handling, stumbling over unnoticed obstacles located below (or above), walking down stairs, taking off shoes.

## 1 Introduction

Unilateral Spatial Neglect (USN) is a common neurological syndrome in which patients fail to detect and respond to stimuli presented on the side of the body or the physical and imaginal space contralateral to the hemispheric lesion (Terruzzi et al., 2023); this deficit cannot be attributed to any elemental sensory or motor impairment. The estimated prevalence of USN after unilateral stroke is 30% and it is more common after right brain damage than after left (Esposito et al., 2021).

USN is a heterogeneous syndrome and may present itself in different ways: it may affect different sensory modalities (Van der Stoep et al., 2013), different spatial reference frames (i.e., egocentric vs. allocentric neglect; Buxbaum et al., 2004), and different regions of space (i.e., peri-personal or extra-personal neglect) or visual imagery (Bisiach and Luzzatti, 1978).

Although neglect may often be characterized by a spatial attentional bias in the horizontal dimension, attention may be oriented in three dimensions of space: horizontal, vertical, and radial (Mennemeier et al., 1992). Most studies of patients with USN focused on horizontal spatial dimension (left–right), with left USN being more frequent (43%) than right USN (19%; Osaki et al., 2022); nevertheless, USN may also occur in vertical (upper-lower) and radial (proximal-distal) spatial dimensions. Spatial bias is also described in healthy subjects who when bisecting vertical lines (intersection of the coronal and sagittal planes) demonstrate a slight upward, forward, and leftward bias (pseudo-neglect) (Chapin et al., 2022).

In addition, spatial neglect may occur in several different spatial reference frames, including head- and body-centered (egocentric), environmental-centered, and object-centered (allocentric) (Morris et al., 2020). The term egocentric neglect is used when a patient is inattentive or unaware of the stimuli that are on one side of his or her body or head. In contrast, allocentric neglect is the condition where a patient is inattentive or unaware of a part of an object or objects and this unawareness appears to be unrelated to the position of this object in relation to the patient’s head and body (Turgut et al., 2017).

Vertical neglect is rarely assessed in clinical practice and is little described in the scientific literature. It was first described by Bender and Teuber (1948), who reported two cases of patients who systematically placed the midpoint of vertically presented lines too high. A similar case of “altitudinal neglect” was later reported by Rapcsak et al. (1988), which is often considered the first article about vertical neglect (e.g., Kim et al., 2001; Hromas et al., 2020). From the available literature, it seems that the vertical bias in patients with ABI may concern both the lower part (Ergun-Marterer et al., 2001; Müri et al., 2009) and the upper part of the space (Shelton et al., 1990; Adair et al., 1995). One possible explanation for the bias in the vertical spatial dimension is that ventral occipitotemporal lesions could lead to upper vertical neglect and occipitoparietal lesions to lower vertical neglect (Halligan and Marshall, 1989; Mark and Heilman, 1997; Pitzalis et al., 1997).

Vertical neglect has often been described in combination with horizontal neglect, to the point that some authors suggested using the terms “diagonal neglect” or “quadrantic neglect” (Mark and Heilman, 1997; Osaki et al., 2022), and in co-occurrence with radial neglect.

Radial neglect (i.e., relative to the transverse plane) was first described by Shelton et al. (1990) and can be assessed both within peripersonal space (e.g., paper and pencil line bisection tasks) and between peripersonal and extrapersonal space.

Although the terms “radial,” “vertical,” and “altitudinal” neglect are often used interchangeably, they do not actually describe the same spatial dimension. “Altitudinal” and “vertical” refer to the sagittal plane, whereas “radial” refers to the transverse plane; however, “vertical” is sometimes used interchangeably concerning both axes (e.g., Halligan and Marshall, 1989, 1991). Confusion increases when radial tasks (e.g., bisecting a vertical line on a sheet of paper on the table, with the lines oriented along the intersection of the midsagittal plane of the viewer) are defined as vertical. This is not entirely incorrect, since a vertical line drawn on a piece of paper on the table can be used to measure the vertical allocentric dimension.

**Figure 1:**
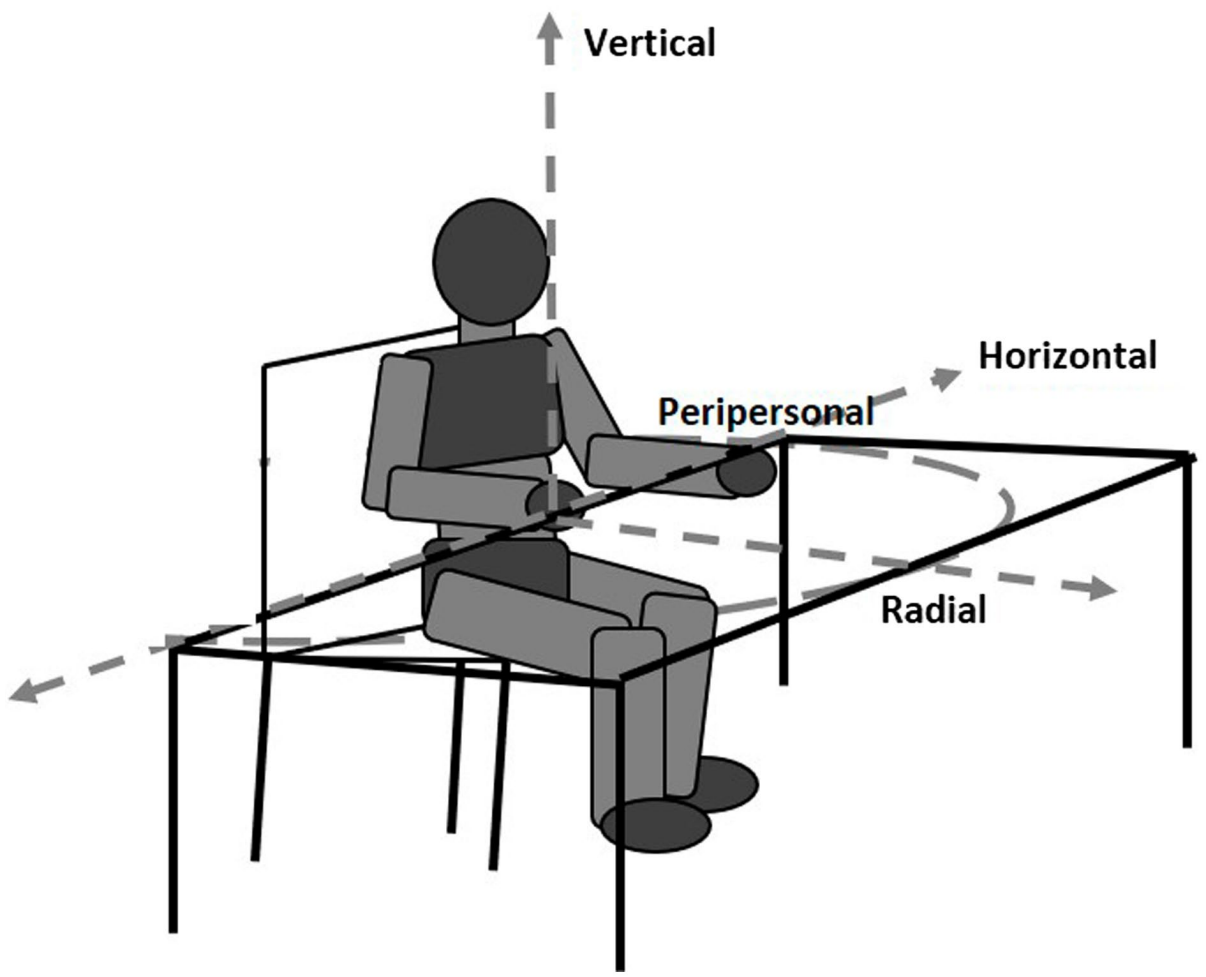
Horizontal, vertical and radial spatial coordinates in visual neglect tasks.

Thus, in these cases, the vertical direction assessed concerns the stimuli and not the subject’s point of view; it could be, therefore, defined as vertical-allocentric neglect, or radial-egocentric neglect (Figure 1).

The presence of vertical and radial spatial neglect is rarely assessed in patients with acquired brain injury (ABI); however, it is reasonable to assume that, as horizontal neglect, it may affect quality of life by predicting longer hospitalization and poorer outcomes in functional recovery (Terruzzi et al., 2023). Visual vertical neglect may also increase the risk of falling and, therefore, the probability of injuries and disabilities in these patients (Julayanont et al., 2019).

Within this context, the main aim of this systematic review is to provide a synthesis of the studies in the literature addressing visual vertical neglect in patients with ABI, with special reference to its clinical manifestations, and etiology and site of lesions.

Another possible aim of this paper is to clarify any differences and common points between vertical and radial neglect. Last, we also aim to suggest potential guidelines in the methods of assessment and treatment of patients with vertical neglect following an ABI.

## 2 Methods

### 2.1 Protocol and registration

This systematic review was registered in the PROSPERO International Prospective Register of Systematic Reviews, registration number CRD42023478713. It was conducted according to the Preferred Reporting Items for Systematic Reviews and Meta-Analyses (PRISMA, Moher et al., 2009).

### 2.2 Eligibility criteria

The focus of this systematic review was to analyze the current literature on vertical spatial neglect in patients with acquired brain injury (ABI). Specifically, we aimed to identify clinical manifestations, assessments, and treatment options.

Peer-reviewed English studies were included if they met the following criteria: (i) patients with ABI (etiology traumatic or vascular or anoxic); we did not select patients with a particular etiology or hemispheric lesion; (ii) presence of vertical spatial neglect (we included patients with visual vertical neglect even if they had no horizontal neglect); (iii) patients over 18 years old; and (iv) specific neuropsychological assessments or treatment for visual vertical neglect were performed. Only studies on visual vertical neglect were eligible for inclusion. We included observational studies such as cross-sectional, case report, and case series studies.

We excluded studies that reported: (i) meta-analyses, reviews or overviews; (ii) book, comments, dissertations; (iii) patients with evidence of horizontal spatial neglect but no evidence of vertical neglect; (iv) vertical neglect due to non-acquired brain injury (e.g., dementia); and (v) not relevant studies to our aims (e.g., studies aimed to indagate verticality judgment but not vertical spatial neglect).

### 2.3 Search strategy and study selection

A systematic literature search was comprehensively conducted on 16 October 2023 in the PsycInfo (PROQUEST), PubMed, and Scopus databases. Search query utilized the term string: (vertical OR altitudinal) AND (neglect OR “unilateral visual inattention” OR hemi-inattention OR hemiagnosia OR hemineglect) AND (diagnosis OR rehabilitation OR treatment). No restrictions or filters were added.

To avoid multiple publication bias, the potential series overlap between studies was evaluated, and if there was overlap (studies referred to the same patients and outcomes), the most relevant study was chosen based on the most informative outcomes (e.g., both vertical and radial neglect), sample size, risk of bias, age of publication, choice of neuropsychological test (better if there was a validate tests).

### 2.4 Screening and data extraction

Article screening (title, abstract, and full-text), data extraction, and quality evaluation were independently conducted by two investigators (NDC and EF) by using a double-blinded approach. Disagreements between reviewers were resolved by discussion. Rayyan software was used to record decisions (Ouzzani et al., 2016).

The extracted information included: (i) publication characteristics; (ii) sample characteristics; (iii) characteristics of vertical neglect (up/down) and associated manifestations (e.g., horizontal neglect, visual field deficit); (iv) injury characteristics (etiology, time post-onset, lesion type, and brain areas); (v) assessment modalities (i.e., paper-and-pencil or computer-based); and (vi) neuropsychological tests used to assess vertical neglect.

### 2.5 Quality assessment

The critical appraisal of the methodological quality of the studies was performed using the appropriate Joanna Briggs Institute (JBI) checklist for each study design employed in the included articles (Moola et al., 2020). The JBI checklists for analytical cross-sectional, case series, and case report study designs were used. According to each study design, the checklist contained each assessment criterion (Supplementary material S1). Each criterion was given a rating of “yes,” “no,” “unclear” or “not applicable.” For cross-sectional and case report studies there are eight criteria, however, for case series are 10. No article was excluded on the basis of these assessments.

## 3 Results

### 3.1 Study selection

**Figure 2:**
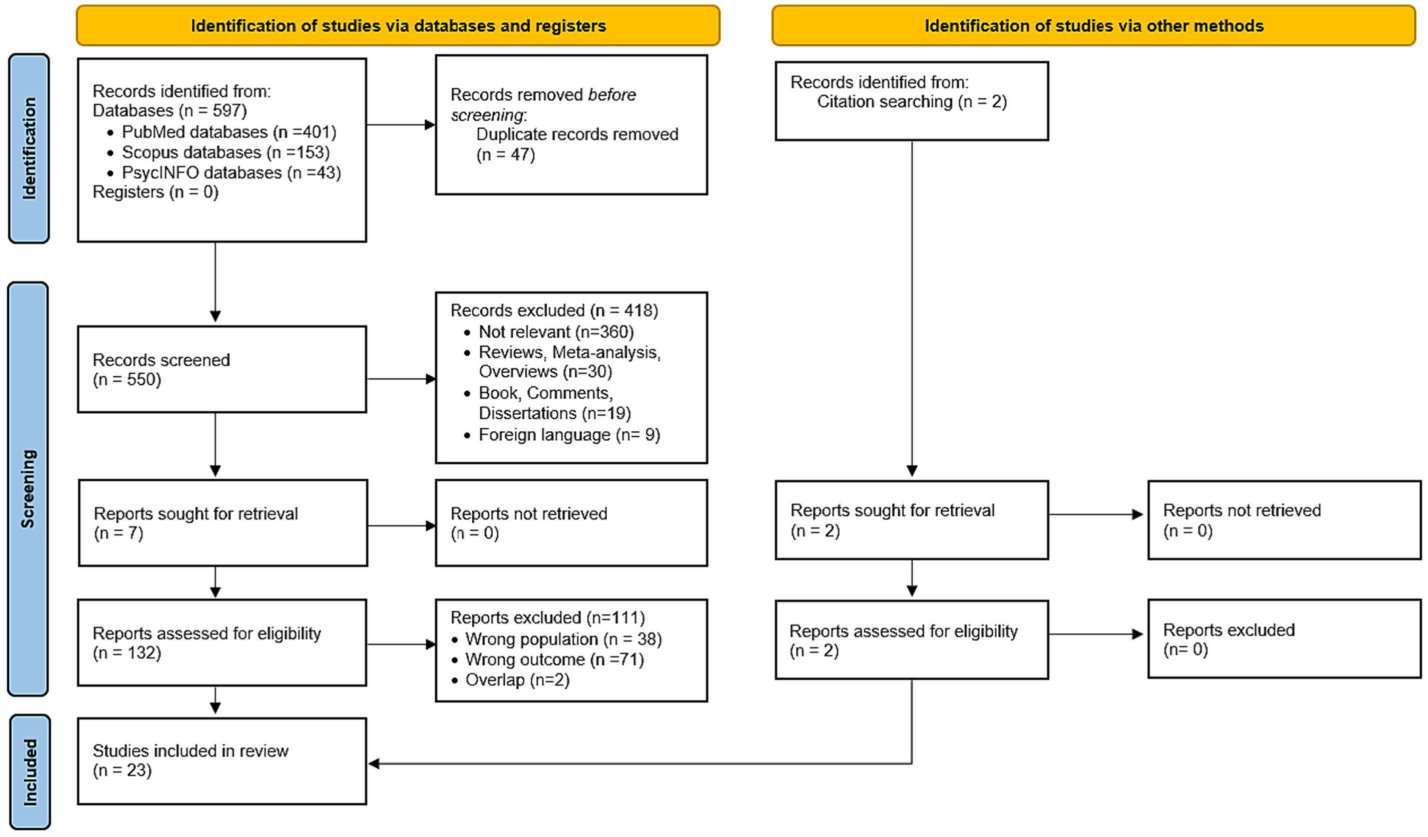
Study selection process according to PRISMA 2020 guidelines.

A total of 550 studies were identified after removing duplicates, and 133 met the criteria for full-text review. Thirty-eight articles were excluded for not reporting an ABI population (e.g., healthy population or dementia patients) or because the participants did not show neglect (e.g., ABI patients without neglect); 71 were excluded because they presented wrong outcomes (e.g., absence of vertical neglect) and 2 reported overlapped data. We included after full-text screening 23 studies (Figure 2).

The included studies are: 10 cross-sectional (Halligan and Marshall, 1989; Kageyama et al., 1994; Làdavas et al., 1994a,b; Pitzalis et al., 1997, 2001; Müri et al., 2009; Cazzoli et al., 2011; Moreh et al., 2014; Osaki et al., 2022); 10 case reports (Butter et al., 1989; Shelton et al., 1990; Halligan and Marshall, 1991; Mennemeier et al., 1992; Nichelli et al., 1993; Adair et al., 1995; Kori and Geldmacher, 1999; Ergun-Marterer et al., 2001; Morris et al., 2020; Numao et al., 2021); and 3 case series (Burnett-Stuart et al., 1991; Halligan and Marshall, 1993; Cappelletti et al., 2007). They were published between 1989 and 2022.

### 3.2 Risk of bias

The quality assessment tool is designed to evaluate the risk to the target population, the recruitment procedures, the description of the subjects and the setting, the methods used for identification, the measurement of exposure, the condition and outcomes, and the appropriate analysis and treatment procedures. The methodological quality of the included studies is summarized in Table 1.

**Table 1:** JBI critical appraisal checklist for analytical case report, cross-sectional, and case series studies.

Case report								
Reference	Q1	Q2	Q3	Q4	Q5	Q6	Q7	Q8
Adair et al. (1995)	Y	Y	Y	Y	NA	NA	NA	Y
Butter et al. (1989)	Y	Y	Y	Y	NA	NA	NA	Y
Ergun-Marterer et al. (2001)	Y	Y	Y	Y	NA	NA	NA	Y
Halligan and Marshall (1991)	Y	Y	Y	Y	NA	NA	NA	Y
Kori and Geldmacher (1999)	Y	N	Y	Y	NA	NA	NA	Y
Mennemeier et al. (1992)	Y	Y	Y	Y	NA	NA	NA	Y
Morris et al. (2020)	Y	Y	Y	Y	NA	NA	NA	Y
Nichelli et al. (1993)	Y	Y	Y	Y	NA	NA	NA	Y
Numao et al. (2021)	Y	Y	Y	Y	Y	Y	N	Y
Shelton et al. (1990)	Y	Y	Y	Y	NA	NA	NA	Y

Cross-sectional								
Reference	Q1	Q2	Q3	Q4	Q5	Q6	Q7	Q8
Cazzoli et al. (2011)	N	Y	Y	Y	Y	N	Y	Y
Halligan and Marshall (1989)	N	Y	Y	Y	Y	Y	Y	Y
Kageyama et al. (1994)	Y	Y	Y	Y	Y	Y	Y	Y
Làdavas et al. (1994a)	Y	Y	Y	Y	Y	Y	Y	Y
Làdavas et al. (1994b)	Y	Y	Y	Y	Y	Y	Y	Y
Moreh et al. (2014)	Y	Y	Y	Y	Y	Y	Y	Y
Müri et al. (2009)	Y	Y	N	N	Y	Y	Y	Y
Osaki et al. (2022)	Y	Y	Y	Y	Y	Y	Y	Y
Pitzalis et al. (1997)	Y	Y	Y	Y	Y	Y	Y	Y
Pitzalis et al. (2001)	Y	Y	Y	Y	Y	Y	Y	Y

Case series										
Reference	Q1	Q2	Q3	Q4	Q5	Q6	Q7	Q8	Q9	Q10
Burnett-Stuart et al. (1991)	N	Y	Y	N	N	Y	Y	Y	N	Y
Cappelletti et al. (2007)	N	Y	Y	N	N	Y	Y	Y	Y	Y
Halligan and Marshall (1993)	N	Y	Y	N	N	Y	Y	Y	N	Y

Y, yes; N, No; NA, Not Available.

### 3.3 Participants and descriptive data

In total, 241 ABI participants were selected (mean age = 62.11 years, SD = 13.56 years; female *n* = 112, but we do not have data on the sex of 7 participants) and all of these reported significant impairment in the vertical dimension. The majority of these subjects (94.6% of the participants) showed lower vertical neglect although in 7 studies (Shelton et al., 1990; Burnett-Stuart et al., 1991; Halligan and Marshall, 1993; Kageyama et al., 1994; Adair et al., 1995; Kori and Geldmacher, 1999; Morris et al., 2020) were reported subjects with upper vertical neglect (5.4% of the participants).

All studies showed that horizontal neglect occurred together with vertical neglect except for two where the presence of horizontal neglect is not specified (Butter et al., 1989) or not significant (Adair et al., 1995). Horizontal neglect was always on the left except in two patients with right horizontal neglect (Pitzalis et al., 2001; Morris et al., 2020).

In addition, vertical neglect can also occur alongside radial neglect. In particular, we found a concomitance between lower vertical neglect and near radial neglect in 11 participants (Mennemeier et al., 1992; Kageyama et al., 1994; Ergun-Marterer et al., 2001; Pitzalis et al., 2001; Numao et al., 2021), and a co-occurrence of lower vertical neglect and far radial neglect in 3 participants (Kageyama et al., 1994; Pitzalis et al., 2001). Furthermore, upper vertical neglect can occur along with far radial neglect in 3 participants (Shelton et al., 1990; Kageyama et al., 1994; Adair et al., 1995), and with near radial neglect in only one (Kori and Geldmacher, 1999). Finally, Nichelli et al. (1993) described a case report involving a patient who showed lower and upper vertical neglect (the latter being less severe) associated with left horizontal neglect, left neglect dyslexia, and lower neglect dyslexia.

Finally, visual field deficits are described in 37 participants (Butter et al., 1989; Halligan and Marshall, 1989, 1991, 1993; Shelton et al., 1990; Burnett-Stuart et al., 1991; Nichelli et al., 1993; Kageyama et al., 1994; Kori and Geldmacher, 1999; Ergun-Marterer et al., 2001; Pitzalis et al., 2001; Cappelletti et al., 2007) and ocular motility deficits are present in 3 of them (Shelton et al., 1990; Mennemeier et al., 1992; Adair et al., 1995).

Demographic and clinical characteristics of participants are detailed in Table 2.

**Table 2:** Characteristics of primary studies included in the systematic review.

Authors	Country	Study type	Demographic and clinical characteristics						Injury characteristics				Assessment characteristics	
			n	Age m (SD)	n F	Vertical space neglected	Other spatial neglect manifestations	Visual field deficit	Etiology	Time post onset (months)	Lesion type	Brain areas	Assessment Modality	Cognitive tools
Adair et al. (1995)	USA (Florida)	Case report	1	67	1	Upper	Far radial neglect	N	Ischaemic	2	Cortical	Bilateral inferior occipital and temporal regions	Paper-and-Pencil	Line bisection test
Burnett-Stuart et al. (1991)	UK	Case series	6	69	0	Upper	Left horizontal neglect	Y	Vascular	9	Cortical	Right temporal and parietal regions	Paper-and-Pencil	Line bisection test
				60	1	Upper	Left horizontal neglect	Y	Vascular	24	Cortical	Right temporal and parietal regions	Paper-and-Pencil	Line bisection test
				69	1	Upper	Left horizontal neglect	N	Vascular	3	Cortical	Right frontal and parietal regions	Paper-and-Pencil	Line bisection test
				47	0	Lower	Left horizontal neglect	Y	Vascular	3	Cortical	Right temporal lobe	Paper-and-Pencil	Line bisection test
				54	0	Lower	Left horizontal neglect	Y	Vascular	1	Cortical	Right occipital and temporal regions	Paper-and-Pencil	Line bisection test
				58	0	Lower	Left horizontal neglect	Y	Vascular	4	Cortical	Right temporal and parietal regions	Paper-and-Pencil	Line bisection test
Butter et al. (1989)	USA (Michigan)	Case report	1	38	1	Lower	NA	Y	TBI	6	Cortical	Bilateral dorsal occipital lobes, cuneal and precuneal regions	Multimodal	Visual and tactile/kinesthetic bisection test, auditory bisection test, line drawing test
Cappelletti et al. (2007)	UK	Case series	3	64	1	Lower	Left horizontal neglect	Y	Ischaemic	NA	Cortical and subcortical	Right temporal and occipital lobes and right thalamus	Multimodal	Mental number bisection test, line bisection test, landmark task
				55	0	Lower	Left horizontal neglect	N	Haemorrhagic	NA	Cortical	Right frontoparietal	Multimodal	Mental number bisection test
				69	1	Lower	Left horizontal neglect	N	Ischaemic	NA	Cortical	Right frontoparietal and temporal cortex	Multimodal	Mental number bisection test
Cazzoli et al. (2011)	Switzerland	Cross-sectional	13	54.8 (8.6)	4	Lower left (time-dependent)	Left horizontal neglect	N	Vascular	1.8	Cortical or cortical and subcortical	Right frontal, right parietal, right temporal, right insula, basal ganglia, operculum, thalamus	Computer-based (eye-tracker)	Visual search
Ergun-Marterer et al. (2001)	Austria	Case report	1	27	1	Lower	Left horizontal neglect, near radial neglect	Y	Ischaemic	11	Cortical	Right occipital cortex including the cingulate gyrus and cuneus partially	Multimodal	Line bisection test, stick bisection test
Halligan and Marshall (1989)	UK	Cross-sectional	23	60	12	Lower	Left horizontal neglect	Y (*n* = 14) N (*n* = 9)	Vascular	78.9	NA	Right hemisphere	Paper-and-Pencil	Line cancelation task
Halligan and Marshall (1991)	UK	Case report	1	54	0	Lower	Left horizontal neglect	Y	Ischaemic	1	Cortical and subcortical	Right occipital and inferior temporal lobes, posterior limb of the right internal capsule	Paper-and-Pencil	Line bisection test, point the center of figures
Halligan and Marshall (1993)	UK	Case series	5	69	0	Upper	Left horizontal neglect	Y	Vascular	6	Cortical	Right temporoparietal	Paper-and-Pencil	Line bisection test
				45	0	Upper	Left horizontal neglect	Y	Vascular	15	Cortical	Right occipitoparietal	Paper-and-Pencil	Line bisection test
				69	1	Upper	Left horizontal neglect	N	Vascular	3	Cortical	Right frontoparietal	Paper-and-Pencil	Line bisection test
				60	1	Upper	Left horizontal neglect	Y	Vascular	20	Cortical	Right temporoparietal	Paper-and-Pencil	Line bisection test
				58	0	Lower	Left horizontal neglect	Y	Vascular	4	Cortical	Right temporoparietal	Paper-and-Pencil	Line bisection test
Kageyama et al. (1994)	Japan	Cross-sectional	7	77	NA	Upper	Left horizontal and far radial neglect	Y	Ischaemic	NA	Cortical and subcortical	Right frontal, temporal and parietal regions, basal ganglia	Paper-and-Pencil	Line bisection test
				73	NA	Upper	Left horizontal and far radial neglect	N	Ischaemic	NA	Cortical	Right frontal lobe	Paper-and-Pencil	Line bisection test
				74	NA	Lower	Left horizontal and far radial neglect	N	Ischaemic	NA	Cortical and subcortical	Right frontal lobe, basal ganglia	Paper-and-Pencil	Line bisection test
				73	NA	Lower	Left horizontal and near radial neglect	Y	Ischaemic	NA	Cortical	Right frontal, parietal and temporal regions	Paper-and-Pencil	Line bisection test
				76	NA	Lower	Left horizontal neglect	Y	Ischaemic	NA	Cortical	Right occipital lobe	Paper-and-Pencil	Line bisection test
				79	NA	Lower	Left horizontal neglect	N	Ischaemic	NA	Cortical and subcortical	Right frontal, temporal and parietal regions, basal ganglia	Paper-and-Pencil	Line bisection test
				80	NA	Lower	Left horizontal neglect	Y	Haemorrhagic	NA	Cortical	Right temporal, parietal and occipital regions	Paper-and-Pencil	Line bisection test
Kori and Geldmacher (1999)	USA (Ohio)	Case report	1	69	0	Upper	Left horizontal and near radial neglect	Y	Ischaemic	3 (days)	Cortical and subcortical	Posterior limb of the right internal capsule extending into the tip of the mesial temporal lobe, medial tip of the lentiform nucleus, anterior thalamus, and mesencephalon	Paper-and-Pencil	Line bisection test
Làdavas et al. (1994a)	Italy	Cross-sectional	14	62.1 (12.8)	2	Lower	Left horizontal neglect	N	Vascular	15.3	Cortical and subcortical	Right frontal lobe, right parietal lobe, right temporal lobe, and deep	Computer-based	Posner task
Làdavas et al. (1994b)	Italy	Cross-sectional	11	69.4 (8.5)	4	Lower	Left horizontal neglect	N	NA	12.5	Cortical and subcortical	Right temporal lobe, right parietal lobe, and deep	Computer-based	Posner task
Mennemeier et al. (1992)	USA (Florida)	Case report	1	41	1	Lower	Left horizontal and near radial neglect	N	Vascular	NA	Cortical	Bilateral parieto-occipital region: left angular gyrus, left lateral occipital gyrus; right inferior angular gyrus, posterior portion of the right middle temporal gyrus, the lateral right occipital gyrus	Multimodal	Visual and tactile bisection test
Moreh et al. (2014)	Israel	Cross-sectional	14	61 (12)	8	Lower	Left horizontal neglect	N	Vascular	40	Cortical and subcortical	Right hemisphere: insula, putamen, globus pallidus, transverse temporal gyrus of Heschl, superior temporal gyrus, temporal pole, middle temporal gyrus, supramarginal gyrus, angular gyrus, hippocampus and parahippocampal gyrus	Computer-based	Verbal memory task
Morris et al. (2020)	USA (Florida)	Case report	1	72	0	Upper	Right horizontal neglect	N	Ischaemic	1 (day)	Cortical	Right temporal lobe, right inferior parietal lobe	Paper-and-Pencil	Line bisection test, Cancelation task
Müri et al. (2009)	Switzerland	Cross-sectional	15	55.5 (9)	4	Lower	Left horizontal neglect	NA	Vascular	2	NA	Right hemisphere	Computer-based (eye-tracker)	Visual search
Nichelli et al. (1993)	Italy	Case report	1	25	0	Lower (more severe) and upper (less severe)	Left horizontal neglect, left neglect dyslexia, lower neglect dyslexia	Y	TBI	4	Cortical and subcortical	Right temporal and occipital lobe, right basal forebrain	Paper-and-Pencil	Line cancelation task, line bisection test, Bells test, Single word/non-word reading
Numao et al. (2021)	Japan	Case report	1	68	0	Lower	Left horizontal and near radial neglect	NA	Ischaemic	10 (days)	Subcortical	Right corona radiate	Computer-based (virtual reality)	Visual search
Osaki et al. (2022)	Japan	Cross-sectional	20	69 (13)	12	Lower left	Left horizontal neglect	NA	Ischaemic (*n* = 11) Haemorrhagic (*n* = 9)	2	Cortical and subcortical	Right hemisphere: frontal, temporal, parietal and occipital regions; basal ganglia, internal capsula, Insula, right thalamus, corona radiate	Paper-and-Pencil and Computer-based	Bells test, apple test, modified Posner task
Pitzalis et al. (1997)	Italy	Cross-sectional	70	72 (8.9)	39	Lower	Left horizontal neglect	NA	Vascular (*n* = 69), neoplastic (*n* = 1)	5.6	NA	Right hemisphere	Paper-and-Pencil	Line cancelation task
			16	69 (8.1)	7	Lower left (latency)	Left horizontal neglect	N	Vascular	5	NA	Right hemisphere	Paper-and-Pencil and Computer-based (VEPs)	Line cancelation task, visual stimulation
Pitzalis et al. (2001)	Italy	Cross-sectional	14	69.4 (6.4)	7	Lower near radial space^2^	Left horizontal neglect		Vascular	5.3	Cortical and subcortical	Right hemisphere	Computer-based	Motor line bisection, Perceptual line bisection
		Case series^1^	8	75	1	Lower (far radial space)	Left horizontal neglect, greater for stimuli in far space than for near space	N	Ischaemic	3	Cortical	Right frontal, temporal and parietal regions	Computer-based	Motor line bisection
				63	1	Lower (near radial space)	Left horizontal neglect, greater for stimuli in far space than for near space	Y	Haemorrhagic	3	Cortical	Right temporal and parietal regions	Computer-based	Motor line bisection, Perceptual line bisection
				63	0	Lower (near radial space)	Left horizontal neglect, greater for stimuli in far space than for near space	Y	Ischaemic	4	Cortical and subcortical	Right temporal and parietal regions, basal ganglia, corona radiate	Computer-based	Perceptual line bisection
				74	1	Lower (near radial space)	Left horizontal neglect, greater for stimuli in far space than for near space	N	Ischaemic	11	Cortical	Right temporal and occipital regions	Computer-based	Motor line bisection
				69	0	Lower (near radial space)	Left horizontal neglect, greater for stimuli in near space than for far space	N	Ischaemic	1	Cortical	Right frontal and temporal regions	Computer-based	Motor line bisection, Perceptual line bisection
				72	0	Lower (near and far radial space)	Left horizontal neglect, greater for stimuli in near space than for far space	N	Ischaemic	8	Cortical	Right temporal and parietal regions	Computer-based	Motor line bisection, Perceptual line bisection
				68	0	Lower (near radial space)	Left horizontal neglect, greater for stimuli in near space than for far space	N	Ischaemic	1	Cortical	Right frontal, temporal and parietal regions	Computer-based	Motor line bisection
				72	0	Lower (near radial space)	Left horizontal neglect, greater for stimuli in far space than for near space	Y	Ischaemic	2	Cortical	Left frontal, temporal, parietal and occipital regions	Computer-based	Perceptual line bisection
Shelton et al. (1990)	USA (Florida)	Case report	1	66	0	Upper	Left and right (less severe) horizontal neglect, far radial neglect	Y	Ischaemic	6	Cortical and subcortical	Bilateral inferior temporal lobe (fusiform and lingular gyri) and deep occipital lobe	Multimodal	Line cancelation task, copy task, line bisection test, tactuomotor line bisection task

^1^The authors also report individual patient data. ^2^Errors in bisecting lines in lower space was modulated by the radial dimension. ABI, Acquired Brain Injury; m, mean; N, no visual field deficit; NA, Not Available; SD, standard deviation; TBI, Traumatic Brain Injury; VEPs, Visual Evoked Potentials; Y, visual field deficit.

### 3.4 ABI characteristics

#### 3.4.1 Etiology

The majority of the selected studies reported patients with vascular etiology, except in two studies where the etiology was traumatic (Butter et al., 1989; Nichelli et al., 1993). The studies on non-vascular patients did not show different characteristics compared to the rest of the selected studies. The most represented vascular etiology was ischaemic (*n* = 32 participants) and the haemorrhagic pathology was described in 12 participants.

The time post-onset was very heterogeneous with a range from 3 days to 78.9 months (Table 2).

#### 3.4.2 Site of brain injury

The vast majority of selected studies reported a unilateral right brain lesion that could affect either cortical or subcortical regions or both. The most represented is the right cortical lesion. Instead, only 4 studies (Butter et al., 1989; Shelton et al., 1990; Mennemeier et al., 1992; Adair et al., 1995) reported bilateral lesions, and one study (Pitzalis et al., 2001) described a patient with extensive left cortical lesion with right horizontal neglect. In addition, in only one study there is a lesion confined to subcortical regions (Numao et al., 2021).

However, in examining the selected studies that reported inclusion criteria for sample selection (i.e., cross-sectional and case series), we found that the majority of them used right brain lesions and vascular etiology as inclusion criteria (e.g., Làdavas et al., 1994a; Müri et al., 2009; Osaki et al., 2022). Only three studies included patients with both right- and left-brain injuries (Halligan and Marshall, 1989; Kageyama et al., 1994; Pitzalis et al., 2001).

The lesion sites were very heterogeneous in the selected articles, but the most representative lesions involved the temporal lobe, which was affected in 17 studies; the occipital lobe was affected in 13 studies, the parietal lobe in 13 studies, and the frontal lobe in 8 studies. Subcortical lesions mainly affected the basal ganglia, internal capsule, corona radiate, and thalamus.

Details on the characteristics of the injuries suffered by participants are shown in Table 2.

### 3.5 Measurement methods of vertical neglect

In the selected studies, vertical neglect was measured employing a wide variety of tools that can be grouped into three categories: paper-and-pencil, computer-based, and multimodal assessments.

#### 3.5.1 Paper-and-pencil

Paper-and-pencil assessments consisted of tasks where the stimuli were on paper usually positioned 30 centimeters from the subject and comprised line bisection tasks, cancelation tasks, line drawing, copy of drawing, landmark, and point the center of figures tasks, and single word reading.

This method of assessing vertical neglect is the most representative among the selected studies and was used as the only method in 11 studies (Halligan and Marshall, 1989, 1991, 1993; Shelton et al., 1990; Burnett-Stuart et al., 1991; Nichelli et al., 1993; Kageyama et al., 1994; Adair et al., 1995; Pitzalis et al., 1997; Kori and Geldmacher, 1999; Morris et al., 2020) and in addition to multimodal assessment in 5 studies (Butter et al., 1989; Shelton et al., 1990; Mennemeier et al., 1992; Ergun-Marterer et al., 2001; Cappelletti et al., 2007) or computer-based assessment in 2 studies (Pitzalis et al., 1997; Osaki et al., 2022) (see Table 2). The description of the tests utilized is reported in Table 3, along with the corresponding study.

**Table 3:** Description of the tools used to assess vertical neglect.

Paper-and-pencil		
Line bisection test	Participants are required to mark the midpoint of several black lines printed on a A4 sheet	Adair et al. (1995), Burnett-Stuart et al. (1991), Cappelletti et al. (2007), Ergun-Marterer et al. (2001), Halligan and Marshall (1991), Halligan and Marshall (1993), Kageyama et al. (1994), Kori and Geldmacher (1999), Morris et al. (2020), Nichelli et al. (1993), Shelton et al. (1990)
Cancelation test (Line cancelation task; Bells test; Apple test)	Participants are required to cross out all the targets on a A4 sheet	Halligan and Marshall (1989), Morris et al. (2020), Nichelli et al. (1993), Osaki et al. (2022), Pitzalis et al. (1997), Shelton et al. (1990)
Line drawing test	Participants are presented a single vertical line on a A4 sheet; they are asked to put the tip of the pencil at the end of the line and to draw an equally long line in the opposite direction to the one presented	Butter et al. (1989)
Copy of drawing	Participant are asked to copy different drawings with varying degrees of difficulty	Shelton et al. (1990)
Landmark task	Participants are required to judge the position of a mark on a line (i.e., left/right/higher/lower of the midpoint)	Cappelletti et al. (2007)
Point the center of figures	Participants are required to mark the center of figures (squares or circles) individually drawn on A4 sheet	Halligan and Marshall (1991)
Single word reading	Participants are required to read a list of words and non-words in their native language	Nichelli et al. (1993)
Computer-based		
Visual search (eye-tracker)	Participants are required to search for an embedded target object in a photograph	Cazzoli et al. (2011)
Free visual exploration task (eye-tracker)	Participants were instructed to freely explore different naturalistic color photographs of everyday scenes	Müri et al. (2009)
Visual memory task	Participants are shown groups of four pictures of everyday objects; then they are asked to free recall the just seen objects (recall task). 8 min after, participants are shown new and previously presented object; they are asked to report if each item was presented before (recognition task)	Moreh et al. (2014)
Modified Posner task	Participants are required to fix a fixation point in the middle of the screen. A cueing task (an arrow pointing upper left, upper right, lower left, or lower right) appears; then one target is displayed in valid (i.e., the same position pointed by the cue) or invalid position	Làdavas et al. (1994a,b) and Osaki et al. (2022)
Visual stimulation (VEPs)	Participants sit in front of a monitor and are instructed to keep their eyes fixed on the stimuli, while different portions of the visual field are stimulated	Pitzalis et al. (1997)
Motor line bisection	Participants are instructed to use a laser pointer to indicate the center of different lines projected on a screen	Pitzalis et al. (2001)
Perceptual line bisection	Participants are required to say whether a mark is on the right or left of the midline of different lines projected on a screen	Pitzalis et al. (2001)
Virtual reality visual search	Participants wear a head-mounted virtual reality display; they are asked to move their head and neck when they perceive a randomly appearing stimulus (i.e., balloon)	Numao et al. (2021)
Multimodal		
Visual and tactile/kinesthetic bisection test	Participants are required to point the center of several wood rods; they are allowed to see the rods or to touch both ends	Butter et al. (1989), Mennemeier et al. (1992), Shelton et al. (1990)
Stick bisection test	The participant has to determine the midpoint of four wooden sticks placed 30 cm in front of him on a table, approximately centered on the body axis. The sticks are presented horizontally, vertically and upright. He is asked to indicate the midline with a thin marker.	Ergun-Marterer et al. (2001)
Auditory bisection test	Participants are asked to bisect, with their eyes closed, the apparent distance between two clicks one of which is presented above their head, the other below	Butter et al. (1989)
Mental number bisection test	Participants are asked to say which number is in the middle of two orally presented numbers	Cappelletti et al. (2007)

VEPs, Visual Evoked Potentials.

#### 3.5.2 Computer-based

Computer-based assessments were utilized in 9 studies and consisted of tasks administered through a computer screen (Làdavas et al., 1994a,b; Pitzalis et al., 2001; Moreh et al., 2014; Osaki et al., 2022) or other technology such as an eye-tracker system (Müri et al., 2009; Cazzoli et al., 2011), virtual reality (Numao et al., 2021) or electrophysiological tests (Pitzalis et al., 1997). Table 3 provides a detailed description of the computer-based tests used in the selected studies.

#### 3.5.3 Multimodal assessment of vertical neglect

Some selected studies used a multimodal assessment to measure vertical neglect, consisting of a visual and tactile/kinesthetic bisection task (Butter et al., 1989; Shelton et al., 1990; Mennemeier et al., 1992; Ergun-Marterer et al., 2001), or an auditory bisection task (Butter et al., 1989), or a mental number bisection task (Cappelletti et al., 2007) in addition to a paper-and-pencil task (e.g., bisection task). It was applied in 4 studies. A detailed description of the multimodal assessment tests that were used in the selected studies is given in Table 3.

#### 3.5.4 Vertical vs. radial neglect

In the reviewed studies, some paper-and-pencil tasks used to assess vertical neglect (e.g., line bisection or cancelation tasks) measured the vertical allocentric component of the stimuli, i.e., the radial component, taking the subject’s egocentric coordinates as reference. Specifically, 11 studies (Halligan and Marshall, 1989, 1991, 1993; Shelton et al., 1990; Burnett-Stuart et al., 1991; Nichelli et al., 1993; Kageyama et al., 1994; Pitzalis et al., 1997; Ergun-Marterer et al., 2001; Cappelletti et al., 2007; Morris et al., 2020; Osaki et al., 2022) utilized, solely or partially, paper-and-pencil tasks that assess the radial (vertical allocentric) neglect and not the vertical (up-down based on egocentric space) neglect. On the other hand, computer-based tasks always evaluate the vertical (egocentric) space (Table 2).

Finally, 8 selected studies (Shelton et al., 1990; Mennemeier et al., 1992; Kageyama et al., 1994; Adair et al., 1995; Kori and Geldmacher, 1999; Ergun-Marterer et al., 2001; Numao et al., 2021; Osaki et al., 2022) evaluated both vertical and radial dimensions and one of them reported results considering both dimensions together (Pitzalis et al., 2001).

### 3.6 Treatment

Of the 22 studies reviewed, only one addressed the treatment of vertical neglect. Numao et al. (2021) developed a virtual reality-based method that randomly generates balloons in the left–right, up-down, and near-far space on a monitor using a head-mounted display. The patient’s task was to perceive these appearing balloons. This method was used to detect mild neglect that was not detected by conventional paper-and-pencil assessments and as a treatment for unilateral spatial neglect. After virtual reality (VR) treatment, the patient showed a reduction in the time taken to perceive the appearing balloons, especially in the upper left space, suggesting a possible therapeutic effect; however, there were poor improvements in the lower area.

## 4 Discussion

To the best of our knowledge, this is the first review study that addressed vertical neglect, specifically the vertical neglect in ABI. We aimed to identify the main characteristics of vertical neglect after ABI, the diagnostic tools used, and the treatment options. We also proposed a clarification of the manifestations and characteristics of vertical and radial neglect.

In the 23 articles reviewed, we found that the lower space was more compromised than the upper space, that vertical neglect manifestations occurred together with horizontal neglect, and that they could also occur with compromise of the radial space, with the near radial being more common. The most frequent etiology is vascular, particularly ischaemic. The brain regions affected are very heterogeneous and include both cortical and subcortical areas and all lobes, although the temporal lobe is the most affected. Paper and pencil tasks are the most commonly used diagnostic tools to identify vertical neglect, although in recent years there has been an increase in the use of computer-based tasks (Moreh et al., 2014; Numao et al., 2021; Osaki et al., 2022). There is still limited experience in treating vertical neglect, and of the results that have been found, only one study deals with treatment (Numao et al., 2021).

### 4.1 Lower and upper neglect

The findings reported in this systematic review showing that inferior vertical space is more commonly impaired in ABI patients (Halligan and Marshall, 1989; Mennemeier et al., 1992; Pitzalis et al., 2001) but we also found deficits in upper vertical space.

Experimental and clinical evidence suggests the existence of separate neural systems involved in the distribution and manipulation of information along spatial dimensions.

Visual information from the retina is transmitted to the primary visual cortex in the occipital lobes. From here this pathway divides into two: a ventral pathway that runs within the temporal lobes; and a dorsal pathway that runs into the parietal lobes (Adair et al., 1995; Chieffi et al., 2018).

The ventral occipitotemporal pathway carries visual information from the inferior portion of the retina, which receives afferent information from the superior portion of the visual field, and the dorsal occipitoparietal pathway carries information from the superior portion of the retina, which receives information from the inferior portion of the visual field (Chieffi et al., 2018).

Studies in brain-damaged patients have suggested that the ventral occipitotemporal visual network mediates processing in upper visual space (Previc, 1990; Shelton et al., 1990; Adair et al., 1995; Drain and Reuter-Lorenz, 1996) and the dorsal occipitoparietal visual network mediates attention in lower visual space (Rapcsak et al., 1988; Drain and Reuter-Lorenz, 1996; Chieffi et al., 2017).

Drain and Reuter-Lorenz (1996) suggested that the two systems exert mutual inhibitory control over the orientation of attention. That is, occipitotemporal activity directing attention to upper space would override occipitoparietal activity directing attention to lower space. An injury to the occipitoparietal regions could lead to a simultaneous disinhibition of occipitotemporal activity and an upward orientation. Conversely, the occipitotemporal injury would result in the disinhibition of occipitoparietal activity and a downward orientation bias (Drain and Reuter-Lorenz, 1996).

The results of this systematic review show that the presence of occipitoparietal injury can lead to inferior space neglect (Rapcsak et al., 1988; Butter et al., 1989; Mennemeier et al., 1992), whereas occipitotemporal damage can lead to superior space neglect (Shelton et al., 1990; Adair et al., 1995; Morris et al., 2020). However, some participants showed inferior space neglect after a temporal lesion (Burnett-Stuart et al., 1991; Halligan and Marshall, 1991; Nichelli et al., 1993; Pitzalis et al., 2001; Cappelletti et al., 2007) and superior space neglect after a parietal lesion (Burnett-Stuart et al., 1991; Halligan and Marshall, 1993).

The attentional functions of these association areas of the parietal cortex depend on input from several other areas of the brain (Heilman et al., 2000). Therefore, a lesion in the subcortical white matter below the parietal and temporal lobes could affect attentional functions related to the ventral and dorsal pathways.

Another possible explanation lies in the lateralization of the lesion. Some evidence of neglect, manifested by a spatial attentional bias, may be caused by a reduction in attention to the contralesional hemispace by the injured hemisphere (Mesulam, 1981), or by disinhibition of the opposite non-injured hemisphere and an increase in the distribution of attention to ipsilesional space (Kinsbourne, 1993). As in the case of horizontal neglect in which each hemisphere primarily attends to the contralateral egocentric hemispace, it may be possible that while the left hemisphere mediates attention toward the body and downward, and the right hemisphere away from the body and upward (Heilman et al., 1995; Morris et al., 2020).

Many left-hemisphere-mediated visual activities, such as reading and writing, are performed below eye level, in lower space. In contrast, many right-hemisphere-mediated visual tasks, such as face recognition or orientation, are performed above eye level, in upper space.

Furthermore, from an evolutionary point of view, the lower visual field is more closely associated with peripersonal space, where the hands interact with objects, tools, and food, whereas the upper visual field is more closely associated with extrapersonal space, where stimuli are distant and high precision of visual movement performance is not required (Previc, 1990).

Therefore, a possible right hemispheric dominance may mediate spatial attention and be responsible for the upward bias (Morris et al., 2020) but it does not explain the downward neglect shown by patients. Further studies are required to investigate the role of each hemisphere in vertical neglect and to determine the laterality and precise location of lesions.

### 4.2 Co-occurrence in vertical neglect

We found that vertical bias may occur in conjunction with a deficit in radial space, according to the findings of this systematic review and previously reported in the literature (Chieffi et al., 2019; Julayanont et al., 2019; Pisanuwongrak et al., 2020). The vertical and radial tasks (e.g., line bisection tasks) may overlap in part; indeed, a classic pencil-and-paper line bisection task, where participants are asked to mark the center of a line placed on a sheet of paper about 30 cm from the participant, could be either a radial egocentric (head-or-body-centered coordinate) or a vertical allocentric (object-centered coordinate) task. Several studies selected in this systematic review (Halligan and Marshall, 1989, 1991, 1993; Nichelli et al., 1993; Kageyama et al., 1994; Pitzalis et al., 1997; Ergun-Marterer et al., 2001; Osaki et al., 2022) use paper-and-pencil tasks and thus measure the egocentric radial/allocentric vertical dimension. Furthermore, when people perform the radial line bisection task, in addition to moving their eyes downward, they often flex their necks and thus a radial line, at least in part, might be perceived as a vertical line. For this reason, there might have been significant differences in the way in which the images of the central and lateral lines were projected onto the retina. In addition, when the radial line is positioned along the midsagittal plane, the gaze crosses the line perpendicularly, and the image of the distal part is projected onto the inferior retina field, which then projects to the occipitotemporal ventral visual attention stream. In contrast, the image of the proximal portion is projected onto the superior retina field, and this portion of the retina projects to the occipitoparietal dorsal visual attentional stream.

Instead, in the bisection of the vertical line, the upper part of the line projects to the inferior retina and is transmitted to the ventral attentional pathway, while the lower part projects to the superior retina and is transmitted to the dorsal pathway. Thus, both retinotopic and spatiotopic factors may have contributed to the vertical and distal bias. Support for this hypothesis comes from the work of Geldmacher and Heilman (1994) who found that when healthy subjects are asked to bisect lines below eye level, they deviate distally. However, this radial deviation is not observed when these radial lines are placed above eye level.

Previous studies on patients with ABI (Rapcsak et al., 1988; Butter et al., 1989; Shelton et al., 1990; Mennemeier et al., 1992; Adair et al., 1995) have suggested that the occipitoparietal stream directs attention to near space, whereas the occipitotemporal stream directs attention to far space. Therefore, an occipitoparietal brain lesion can distort attentional orientation toward far and upper space, and an occipitotemporal brain lesion can produce a distortion toward near and lower space.

Contrary to some findings in the literature (Mennemeier et al., 1992; Mark and Heilman, 1997; Chieffi et al., 2019; Pisanuwongrak et al., 2020), which suggest a possible overlap of neural networks important for mediating attention in proximal/inferior and distal/superior space, we have found that near/lower and far/upper do not always co-occur. Indeed, the presence of manifestations of lower neglect and deficit for both far (Kageyama et al., 1994; Pitzalis et al., 2001) and near (Mennemeier et al., 1992; Kageyama et al., 1994; Ergun-Marterer et al., 2001; Pitzalis et al., 2001; Numao et al., 2021) space, and of upper neglect and deficit for both far (Shelton et al., 1990; Kageyama et al., 1994; Adair et al., 1995) and near space (Kori and Geldmacher, 1999) suggests that processing of vertical and radial space may be partially independent.

In addition, our results show that horizontal neglect almost always co-occurs with vertical neglect. This evidence might suggest considerable overlap in the mechanisms controlling vertical and horizontal spatial processing, but several studies, that have examined leftward and upward biases in the same participants (Shelton et al., 1990; Halligan and Marshall, 1991, 1993; Kageyama et al., 1994; Adair et al., 1995; Pitzalis et al., 2001), have shown that there is no reliable correlation between the extent of the biases in each of the dimensions (Nicholls et al., 2004), suggesting the existence of co-occurring but distinct constructs.

Therefore, our results, together with the available evidence, support the view that horizontal, vertical, and radial spatial asymmetries may be driven by only partially independent cognitive and neural mechanisms.

We also observed that some patients in selected studies had visual field defects in addition to vertical neglect. Patients with vertical visual field defects (altitudinal hemianopia), but without manifestations of neglect, in vertical line or bar bisection tasks, tend to place their midline (or bisection) toward the blind field (Kerkhoff, 1993). Contrarily individuals with vertical neglect position their midline away from the neglected field. Given that cancelation tasks are frequently utilized to evaluate vertical neglect, it is crucial to recognize that vertical field defects can produce an opposing effect. Additionally, individuals with oblique visual field defects (quadrantanopia) similarly demonstrate an oblique shift of their bisection or straight ahead into the blind quadrant (Kuhn et al., 2010).

### 4.3 Assessment of vertical neglect

From this systematic review, it emerges that the most used tools to assess vertical neglect are paper-and-pencil tests and in particular line bisection and cancelation tasks. We found also experience with computer-based or VR assessment. As mentioned previously, the nature of the tasks can influence both the behavioral measure and the spatial dimension explored.

The line bisection task is a perceptual-motor task, which mainly involves allocentric coordinates and is therefore mediated by the occipitotemporal system (Chieffi et al., 2017). It primarily involves magnitude estimates that are affected by attentional bias (Lee et al., 2004). Although sensory-attentional bias most often influences performance on the line bisection test, this test requires eye and hand movements; therefore, it may also reveal action-intentional biases. In contrast, cancelation tests require a systematic visual search and require greater levels of exploration, attentional focusing, attentional disengagement, and sustained vigilance compared to the line bisection task (Lee et al., 2004). These activities are primarily mediated by frontal lobes but an injury to the temporal or parietal lobe can reveal impairment in the cancelation task as well as in the bisection lines task (Morris et al., 2020).

Unlike paper-and-pencil tasks, computer-based tasks primarily assess vertical (egocentric) space because the direction of the stimulus to be processed usually coincides with the up-down body-centered spatial frames. In this way, the upper part of the stimulus is transmitted to the ventral visual pathway and the lower part to the dorsal visual pathway.

Finally, the VR method used by Numao et al. (2021) is a promising tool capable of evaluating the different spatial coordinates together and maintaining a certain ecological value. In addition, VR could facilitate the identification of spatial bias in patients with ABI (Numao et al., 2021). Taking into account horizontal, radial, and vertical coordinates, it not only aids in the detection of neglect but also shows potential therapeutic effects in its treatment.

To our knowledge, this is the only study that aims to rehabilitate vertical neglect and the only one that uses virtual reality as a diagnostic and rehabilitative tool in vertical neglect. However, Numao et al. (2021) results must be interpreted critically. They found an improvement in reaction time to the balloon search task. After treatment, awareness in the left space, especially in the upper left, was significantly improved but this awareness was poor in the lower area. This latter result may be due to the authors setting the tilt angle to 10° from the initial position, which may have affected awareness in the lower left space. In addition, the reported improvements could also be due to an iterative learning effect, as the treatment was based on repeated searching for the balloons. Furthermore, this is a case report without a control group and a possible effect of spontaneous remission cannot be excluded. Finally, changes in reaction times measured in this study may not be related to vertical neglect (Hurford et al., 2013). These promising findings should be approached with caution, and additional studies are warranted to validate these preliminary results.

In the last decades, different VR-based protocols have been proposed for the rehabilitation and assessment of neglect. VR may be an innovative and potentially powerful tool, to be used in conjunction with, or as an alternative to, those already widely used in clinical and rehabilitation practice of patients with ABI with USN regardless of the immersion level (Bohil et al., 2011; Pedroli et al., 2015; Salatino et al., 2023).

However, VR is not the only possible therapeutic tool for the treatment of vertical neglect. Treatments that can benefit the clinical manifestations of neglect in the horizontal dimension, such as non-invasive brain stimulation (Kashiwagi et al., 2018; Yang et al., 2023) or visual scanning training (Gammeri et al., 2020), could be promising tools to also improve vertical symptoms.

To date, traditional paper-and-pencil tests may be inadequate for detecting USN symptoms (Rengachary et al., 2009). In fact, paper-and-pencil tools use static, two-dimensional stimuli that are very different from those of a real or virtual environment (i.e., they are not ecological).

Paper-and-pencil tasks typically require a simple visual search in near space and only allow the diagnosis of peripersonal USN (Kim et al., 2010; Aravind and Lamontagne, 2014), whereas neglect can instead present two subtypes, peripersonal and extrapersonal, which can be dissociated (Halligan et al., 2003).

Instead, everyday life requires dynamic responses to relevant stimuli in both personal and extrapersonal space, which change every time (Kim et al., 2010). With VR, it is possible to recreate this dynamism and build more ecological tasks.

Contrary to paper-and-pencil, in computer-based tasks different reaction time gradients have been observed for both static and moving stimuli (Cazzoli et al., 2011), with a progression from the ipsilesional field toward the midline and in the contralesional field (Dvorkin et al., 2007).

Thus, computer-based tasks are generally more sensitive than paper-and-pencil tests (Rengachary et al., 2009; Bonato et al., 2012).

Therefore, from the evidence presented in this study, computerized and VR-based tests could be a valid alternative to traditional paper-and-pencil tasks in the assessment of USN. These new tools could overcome the radial-egocentric and vertical-allocentric problems present in some paper-and-pencil tasks, making the assessment more sensitive to deficits and more ecological.

Furthermore, in the case of VR, they could facilitate the detection of deficits for each spatial dimension simultaneously and promote the study of vertical coordinates, which are usually little studied.

### 4.4 Limitations

This systematic review has some limitations that must be considered.

The first limitation concerns a bias in the selection of the studies. We selected the studies that concern visual vertical neglect in ABI; in some of these (e.g., Kageyama et al., 1994 or Pitzalis et al., 2001), we selected only the patients with signs of vertical neglect. There might be a bias in the selection of studies and patients, which does not allow us to make strong inferences about the relationship between vertical neglect and other neglect types, different lesion territories, or aetiologies. Moreover, most of the selected studies reported right brain injury and/or vascular etiology as inclusion criteria for patient selection. Therefore, it is difficult to make statements about the prevalence of vertical neglect among different aetiologies and lesion sites. However, we described what we observed, that is the co-occurrence of visual vertical neglect following ABI. Future review studies should investigate the phenomenon of vertical neglect more extensively, also including patients with other types of neglect who do not show signs of vertical neglect.

The second limitation involves potential functional deficits in vertical neglect. Given that the vast majority of included patients have both horizontal and vertical neglect, we cannot exclude that is the horizontal neglect which may lead to a functional deficit in everyday life. Conversely, we cannot exclude that it is the weight of vertical neglect that leads to a functional deficit in daily life. More likely, the presence of both, vertical and horizontal neglect, leads to greater functional deficits.

Third, we selected several single-case studies that are particularly effective in detecting peculiar patterns of performances, but the results of which must be taken with particular caution. Therefore, these studies may reduce the overall quality of the studies analyzed.

Fourth patients may omit targets in the lower quadrants in cancelation tasks because they are fatigued but in the included studies, we could not control this possibility. However, some studies demonstrated that fatigue could not affect vertical bias in cancelation task (Robertson and North, 1993; Mark and Heilman, 1997).

A further limitation identified in all studies, except one (Numao et al., 2021) concerns the lack of rehabilitation for vertical neglect.

Moreover, some studies may not have been identified with the search strategy we used. This could be due both to the heterogeneity of the names indicating neglect syndrome and to the fact that some studies could report vertical biases described as radial.

Lastly, it should be noted that comparison between studies may be biased by the heterogeneity of patients with ABI and brain lesions, the variability of clinical manifestations, the small sample size of most studies, and the year of publication, which reflects the differences in terminology regarding vertical bias (e.g., altitudinal) and in the progress in the use of different tools for assessment (computerized devices and VR). Although there was heterogeneity, the results were relatively consistent, suggesting that vertical bias is a manifestation of USN that should be taken into account.

Future studies should focus on the presence of vertical neglect after ABI trying to identify specific patterns of clinical manifestation, resolving the possible role of cerebral hemispheres and lesion sites in the verticality bias. Furthermore, the possible relationship between vertical neglect and functional impairment in everyday life should be investigated with greater systematic rigor.

### 4.5 Conclusion

Vertical neglect is poorly studied in the literature and rarely assessed in clinical practice. Our results suggest that vertical neglect may be underestimated in patients with right hemisphere lesions and should always be assessed, especially in cases where the patient shows signs of horizontal neglect. Furthermore, vertical neglect can lead to important functional limitations in everyday life, such as poor wheelchair handling, stumbling over unnoticed obstacles located below (or above), walking downstairs, and taking off shoes. However, assessing vertical dimensions with paper and pencil, computer-based and VR tasks could help ABI patients to take fewer risks and pay more attention to the neglected space, and could guide the clinician toward more personalized and decisive therapies.

Based on this review it may be important to test for neglect in all three body-cantered directions horizontal, vertical, and, radial.

## Data availability statement

The original contributions presented in the study are included in the article/Supplementary material, further inquiries can be directed to the corresponding author.

## Author contributions

PM: Conceptualization, Data curation, Formal analysis, Investigation, Methodology, Project administration, Resources, Supervision, Validation, Visualization, Writing – original draft, Writing – review & editing. NC: Conceptualization, Data curation, Formal analysis, Investigation, Methodology, Project administration, Resources, Supervision, Validation, Visualization, Writing – original draft. EF: Conceptualization, Data curation, Formal analysis, Investigation, Writing – original draft. AM: Data curation, Validation, Visualization, Writing – review & editing. CeF: Data curation, Validation, Visualization, Writing – review & editing. PA: Formal analysis, Supervision, Validation, Visualization, Writing – review & editing. CiF: Conceptualization, Data curation, Formal analysis, Validation, Visualization, Writing – review & editing. GS: Formal analysis, Supervision, Validation, Visualization, Writing – review & editing. LM: Conceptualization, Formal analysis, Supervision, Validation, Visualization, Writing – review & editing.
