# Supplementary material for: Visual vertical neglect in acquired brain injury: a systematic review
Source: Front Psychol. 2024 Mar 11;15:1360057. doi: 10.3389/fpsyg.2024.1360057 (PMC10962212; doi:10.3389/fpsyg.2024.1360057)
Supplement: Supplementary file 1 [file Table_1.DOCX]

Supplementary Material

# Supplementary Tables

**Assessment risk of bias of primary studies included in the systematic review.**

**JBI Critical Appraisal Checklist for analytical case report studies**

Q1. Were patient’s demographic characteristics clearly described?

Q2. Was the patient’s history clearly described and presented as a timeline?

Q3. Was the current clinical condition of the patient on presentation clearly described?

Q4. Were diagnostic tests or assessment methods and the results clearly described?

Q5. Was the intervention(s) or treatment procedure(s) clearly described?

Q6. Was the post-intervention clinical condition clearly described?

Q7. Were adverse events (harms) or unanticipated events identified and described?

Q8. Does the case report provide takeaway lessons?

| **Case report** | | | | | | | | |
| --- | --- | --- | --- | --- | --- | --- | --- | --- |
| **Reference** | **Q1** | **Q2** | **Q3** | **Q4** | **Q5** | **Q6** | **Q7** | **Q8** |
| Adair et al. (1995) | Yes | Yes | Yes | Yes | NA | NA | NA | Yes |
| Butter et al. (1989) | Yes | Yes | Yes | Yes | NA | NA | NA | Yes |
| Ergun-Marterer et al. (2001) | Yes | Yes | Yes | Yes | NA | NA | NA | Yes |
| Halligan et al. (1991) | Yes | Yes | Yes | Yes | NA | NA | NA | Yes |
| Kori et al. (1999) | Yes | No | Yes | Yes | NA | NA | NA | Yes |
| Mennemeier et al. (1992) | Yes | Yes | Yes | Yes | NA | NA | NA | Yes |
| Morris et al. (2020) | Yes | Yes | Yes | Yes | NA | NA | NA | Yes |
| Nichelli et al. (1993) | Yes | Yes | Yes | Yes | NA | NA | NA | Yes |
| Numao et al. (2021) | Yes | Yes | Yes | Yes | Yes | Yes | No | Yes |
| Shelton et al. (1990) | Yes | Yes | Yes | Yes | NA | NA | NA | Yes |

NA= Not Available

**JBI Critical Appraisal Checklist for analytical cross-sectional studies**

Q1. Were the criteria for inclusion in the sample clearly defined?

Q2. Were the study subjects and the setting described in detail?

Q3. Was the exposure measured in a valid and reliable way?

Q4. Were objective, standard criteria used for measurement of the condition?

Q5. Were confounding factors identified?

Q6. Were strategies to deal with confounding factors stated?

Q7. Were the outcomes measured in a valid and reliable way?

Q8. Was appropriate statistical analysis used?

| **CROSS-SECTIONAL** | | | | | | | | |
| --- | --- | --- | --- | --- | --- | --- | --- | --- |
| **Reference** | **Q1** | **Q2** | **Q3** | **Q4** | **Q5** | **Q6** | **Q7** | **Q8** |
| Cazzoli et al. (2011) | No | Yes | Yes | Yes | Yes | No | Yes | Yes |
| Halligan et al. (1989) | No | Yes | Yes | Yes | Yes | Yes | Yes | Yes |
| Kageyama et al. (1994) | Yes | Yes | Yes | Yes | Yes | Yes | Yes | Yes |
| Làdavas et al. (1994a) | Yes | Yes | Yes | Yes | Yes | Yes | Yes | Yes |
| Làdavas et al. (1994b) | Yes | Yes | Yes | Yes | Yes | Yes | Yes | Yes |
| Moreh et al. (2014) | Yes | Yes | Yes | Yes | Yes | Yes | Yes | Yes |
| Müri et al. (2009) | Yes | Yes | No | No | Yes | Yes | Yes | Yes |
| Osaki et al. (2022) | Yes | Yes | Yes | Yes | Yes | Yes | Yes | Yes |
| Pitzalis et al. (1997) | Yes | Yes | Yes | Yes | Yes | Yes | Yes | Yes |
| Pitzalis et al. (2001) | Yes | Yes | Yes | Yes | Yes | Yes | Yes | Yes |

**JBI Critical Appraisal Checklist for analytical case series studies**

Q1. Were there clear criteria for inclusion in the case series?

Q2. Was the condition measured in a standard, reliable way for all participants included in the case series?

Q3. Were valid methods used for identification of the condition for all participants included in the case series?

Q4. Did the case series have consecutive inclusion of participants?

Q5. Did the case series have complete inclusion of participants?

Q6. Was there clear reporting of the demographics of the participants in the study?

Q7. Was there clear reporting of clinical information of the participants?

Q8. Were the outcomes or follow up results of cases clearly reported?

Q9. Was there clear reporting of the presenting site(s)/clinic(s) demographic information?

Q10. Was statistical analysis appropriate?

| **CASE SERIES** | | | | | | | | | | |
| --- | --- | --- | --- | --- | --- | --- | --- | --- | --- | --- |
| **Reference** | **Q1** | **Q2** | **Q3** | **Q4** | **Q5** | **Q6** | **Q7** | **Q8** | **Q9** | **Q10** |
| Burnett-Stuart et al. (1991) | No | Yes | Yes | No | No | Yes | Yes | Yes | No | Yes |
| Cappelletti et al. (2007) | No | Yes | Yes | No | No | Yes | Yes | Yes | Yes | Yes |
| Halligan et al. (1993) | No | Yes | Yes | No | No | Yes | Yes | Yes | No | Yes |
